# Ultrasound–Microwave Combined Extraction of Novel Polysaccharide Fractions from *Lycium barbarum* Leaves and Their In Vitro Hypoglycemic and Antioxidant Activities

**DOI:** 10.3390/molecules28093880

**Published:** 2023-05-04

**Authors:** Na Quan, Yi-Dan Wang, Guo-Rong Li, Zi-Qi Liu, Jing Feng, Chun-Lei Qiao, Hua-Feng Zhang

**Affiliations:** 1 International Joint Research Center of Shaanxi Province for Food and Health Sciences, Provincial Research Station of Se-Enriched Foods in Hanyin County of Shaanxi Province, College of Food Engineering and Nutritional Science, National Engineering Laboratory for Resource Development of Endangered Crude Drugs in Northwest China, Shaanxi Normal University, Xi’an 710119, China; 2 Yinchuan Market Supervision Administration, Yinchuan 750001, China; 3 Agrarian and Technological Institute, Peoples’ Friendship University of Russia, Moscow 119991, Russia

**Keywords:** *Lycium barbarum* leaves, ultrasound–microwave combined extraction, polysaccharides, antioxidant capacity, hypoglycemic activity

## Abstract

Ultrasound–microwave combined extraction (UMCE), gradient ethanol precipitation, chemical characterization, and antioxidant and hypoglycemic activities of *Lycium barbarum* leaf polysaccharides (LLP) were systematically studied. The optimal conditions for UMCE of LLP achieved by response surface method (RSM) were as follows: microwave time of 16 min, ultrasonic time of 20 min, particle size of 100 mesh, and ratio of liquid to solid of 55:1. Three novel polysaccharide fractions (LLP_30_, LLP_50_, LLP_70_) with different molecular weights were obtained by gradient ethanol precipitation. Polysaccharide samples exhibited scavenging capacities against ABTS and DPPH radicals and inhibitory activities against α-glucosidase and α-amylase. Among the three fractions, LLP_30_ possessed relatively high antioxidant and hypoglycemic activities in vitro, which showed a potential for becoming a nutraceutical or a phytopharmaceutical for prevention and treatment of hyperglycemia or diabetes.

## 1. Introduction

Diabetes is one of the most serious chronic diseases worldwide, mainly caused by insulin deficiency or resistance [1,2]. The typical symptom of diabetes is hyperglycemia, which is possibly harmful to human eyes, kidneys, heart, blood vessels, and nerves. Injection of insulin or oral administration of hypoglycemic agents such as sulfonylureas and biguanides are prevailing strategies to alleviate the disease [3]. However, long-term utility of synthetic drugs may lead to side effects [4]. Therefore, screening hypoglycemic constituents from natural products become a promising way to develop anti-diabetic medicines taking into account the observed toxicological profile of synthetic drugs. Currently, there are numerous natural hypoglycemic products available in the market, most of which are coming from bioactive ingredients of natural plants, such as flavonoids, saponins, and polysaccharides [1,5]. In many cases, natural active components exhibited relatively high hypoglycemic activity and few side effects, which enabled them to be intensively investigated to develop anti-diabetic medicines.

*Lycium barbarum* belongs to the family of Solanaceae. In the Chinese medicinal monographs “Bencao Gangmu (Compendium of Materia Medica)” and “Shennong Bencao Jing (Shennong’s Classic of Materia Medica)”, *Lycium barbarum* leaves are labeled “Di Xian Miao (Seedling of Earth God)”. They are rich in nutrients and bioactive substances, such as proteins, amino acids, vitamins, trace elements, flavonoids, alkaloids, polysaccharides, and terpenes [6]. Usually, Chinese traditional prescription employed the fruits of *Lycium barbarum* to deal with diabetes. For example, the famous anti-diabetic prescription “Kangji Xiaoke Pian (Tablets against Hunger and Thirst)” with National Medicine Permission Number of Z11020440 from Beijing TRT Group (Tong Ren Tang Co., Ltd.) contained *Lycium barbarum* fruits. It is reported that polysaccharides extracted from *Lycium barbarum* fruits display anti-tumor and immunostimulatory activities [7]. Moreover, they improve oral glucose tolerance in diabetic mice by down-regulating the related gene expression in liver [8], indicating their superior hypoglycemic and hypolipidemic capacities. Although *Lycium barbarum* fruits are mostly used to extract polysaccharides, the cost of fruits is expensive, and the fruits are frequently jeopardized by moths and other pests. A great number of recent studies have demonstrated that *Lycium barbarum* leaves are basically consistent with the fruits in terms of nutrients and bioactive substances, and contents of some important constituents (e.g., betaine) in leaves are even higher than those in the fruits [6,9,10]. Moreover, *Lycium barbarum* leaves are relatively cost-effective when compared with the fruits. Thus, *Lycium barbarum* leaves show a great potential for becoming a cheap and abundant source of health-promoting compounds. Nevertheless, there are few reports on extraction of novel polysaccharide fractions with antioxidant and hypoglycemic capacities from *Lycium barbarum* leaves, and their mechanism of action remains unclear. At present, *Lycium barbarum* leaves are mainly used as leafy vegetables or herbal tea, and their intensive processing is greatly lacking [6]. Therefore, it is of great theoretical and practical significance to extract bioactive components from *Lycium barbarum* leaves. Traditionally, hot water extraction (HWE), ultrasonic-assisted extraction (UAE), and microwave-assisted extraction (MAE) are employed to extract polysaccharides from *Lycium barbarum* [11]. However, extraction duration (extraction time) is long while extraction yield is undesirable in many cases. Compared with conventional extraction methods such as hot water extraction, ultrasound–microwave combined extraction (UMCE) usually improves extraction yield of polysaccharides, by which the advantages of ultrasound and microwave were integrated [12,13]. To our knowledge, there is no report on ultrasound–microwave combined extraction of polysaccharides with hypoglycemic and antioxidant properties from *Lycium barbarum* leaves so far.

In this study, bioactive polysaccharides were extracted from *Lycium barbarum* leaves using ultrasound–microwave combined extraction for the first time and were subsequently fractioned according to their molecular weights by gradient ethanol precipitation method. Chemical properties (e.g., molecular weights) of polysaccharide samples were investigated. Moreover, antioxidant capacities of polysaccharide samples were analyzed, and their inhibitory activities against α-glucosidase and α-amylase were studied. The present research will develop a protocol of ultrasound–microwave combined extraction to isolate bioactive polysaccharides from *Lycium barbarum* leaves, which might be used as a potential source of natural antioxidants and as a value-added ingredient in the preparation of functional foods or phyto-pharmaceuticals with anti-diabetic activities.

## 2. Results

### 2.1. Ultrasound–Microwave Combined Extraction of Lycium barbarum Leaf Polysaccharides (LLP)

#### 2.1.1. Effects of Extraction Variables on Extraction Yield of LLP

**Figure 1:**
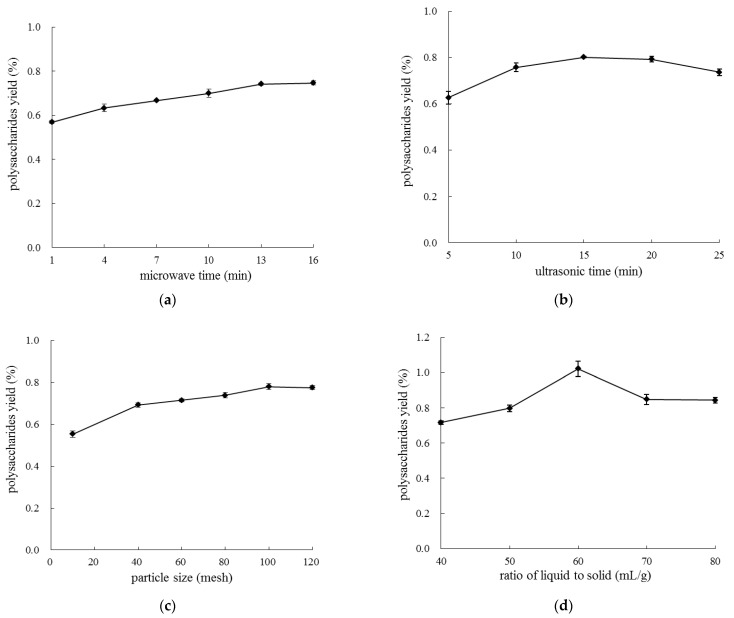
Effects of microwave time (**a**), ultrasonic time (**b**), particle size (**c**), and ratio of liquid to solid (**d**) on extraction yield of LLP.

It can be seen from Figure 1a that extraction yield of LLP increased with the prolongation of microwave time. The yield of polysaccharides increased rapidly from 0.57% to 0.74% during 1~13 min (*p* < 0.05), and it increased slowly after 13 min (*p* > 0.05). The loss of polysaccharides might be attributed to thermal degradation caused by excessive exposure to microwave irradiation [12]. Therefore, microwave time ranging from 10 to 16 min was selected for the following response surface assay.

As shown in Figure 1b, in the range of ultrasonic time from 5 to 15 min, the yield of LLP increased significantly from 0.63% to 0.80% (*p* < 0.05); then, the yield declined when ultrasonic process lasted longer than 15 min (*p* > 0.05). With the extension of treatment time, intensified ultrasound might break the chain of LLP, which would result in the reduction of polysaccharide yield. Thus, ultrasonic time should be maintained in the range of 5 to 25 min.

When particle size of *Lycium barbarum* leaves was 10 mesh (2.00 mm), the yield of polysaccharides was 0.55% (Figure 1c). When particle size was 100 mesh (0.15 mm), the yield of polysaccharides significantly increased to 0.78% (*p* < 0.05), suggesting that small particle size is conducive to polysaccharides dissolution from plant material matrix to extractant. When particle size decreased to 120 mesh (0.12 mm), the increment of the yield became insignificant (*p* > 0.05). The loss of polysaccharide yield occurred when particle size was further diminished, probably because the heat generated during grinding process partially damaged polysaccharides [14]. Thus, particle size ranging from 80 to 120 was selected for the following response surface assay.

As shown in Figure 1d, when the ratio of liquid to solid was set between 40 and 60 mL/g, the yield of polysaccharides increased significantly from 0.72% to 1.00% (*p* < 0.05). However, the yield notably declined in the range of 60 to 70 mL/g (*p* < 0.05), which was possibly attributed to extraction temperature declined with the increase of the solvent volume [12]. This result was consistent with those reported by Zeng et al. (2015) [15]. Therefore, liquid–solid ratio ranging from 40 to 70 mL/g was selected for the response surface experiments.

#### 2.1.2. Optimization of LLP Extraction by Response Surface Method (RSM)

##### Model Fitting and Statistical Analysis

The response surface method is an effective strategy to optimize experimental conditions [16]. RSM mainly used quadratic regression equation for fitting the relationship between multiple factors and multiple response values. The optimal conditions were obtained by regression equation analysis. In order to further optimize the conditions for ultrasound–microwave combined extraction of LLP, Box–Behnken Design (BBD) was carried out based upon the above-mentioned single factor experiments (Table 1 and Table 2). According to the multiple regression analysis, the relationship between dependent and independent variables was expressed as the following second-order polynomial equation:

Y = 1.583 + 0.299A + 0.114B + 0.182C + 0.059D − 0.221B^2^ − 0.219C^2^ − 0.274D^2^

where A, B, C, D, and Y represented microwave time (min), ultrasonic time (min), particle size (mesh), ratio of liquid to solid (mL/g), and extraction yield of polysaccharides (%), respectively.

Usually, response plots based on the fitted polynomial equation were used to visualize the relationships between the response value and experimental level of each factor and to deduce the optimum condition [17]. The absolute value of each coefficient in the equation reflected the degree of influence of various factors on LLP yield. The positive and negative coefficients reflected the direction of influence. The order of the factors affecting the yield was: microwave time > particle size > ultrasonic time > liquid–solid ratio. It can be observed that microwave time, ultrasonic time, particle size, and liquid–solid ratio all showed positive effects. That is to say, LLP yield increased with the improvement of microwave time, ultrasonic time, particle size, and liquid–solid ratio. The equation showed that the coefficient of square of each factor was negative, indicating that three-dimensional (3D) response surface of the model was parabolic, and the maximum value existed clearly.

In order to test the validity of the equation, the model was analyzed by ANOVA (analysis of variance). As shown in Table 3, the result of *F* test (mean square regression: mean square residual is 25.344) was significant (*p* < 0.05), indicating that the selected model was adequate to predict the relationship between the variables and the yields [18]. The correlation coefficient (*R*^2^) of the model was 0.9620, indicating that the selected parameters were significantly related to LLP yield within the range of the experimental variables. Meanwhile, the relatively high adjusted correlation coefficient (*R*^2^_adj_ = 0.9241) demonstrated the reliability of the established model [19]. As a result, the established model is supposed to fit well the practical conditions of microwave time, ultrasonic time, particle size, and ratio of liquid to solid in LLP extraction.

##### Analysis of Response Surface Plots and Contour Plots

**Figure 2:**
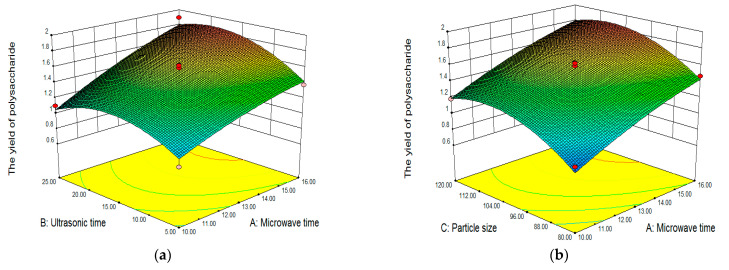

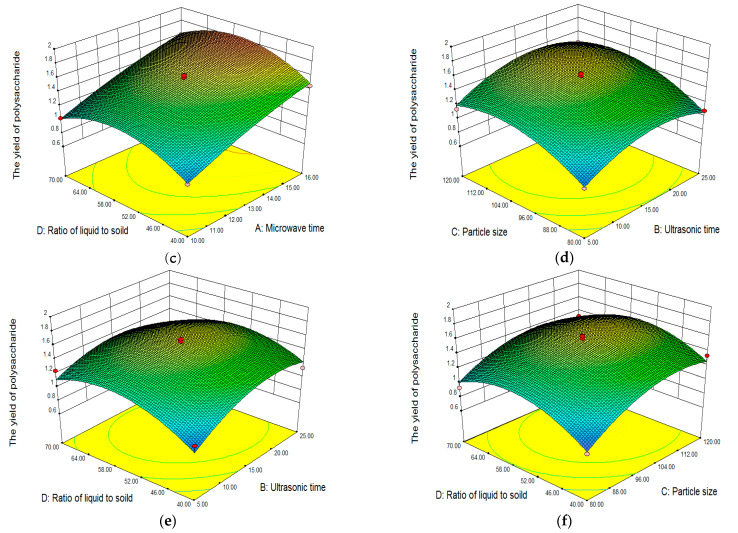
Response surface plots of ultrasound–microwave combined extraction. (**a**) microwave time versus ultrasonic time; (**b**) microwave time versus particle size; (**c**) microwave time versus ratio of liquid to solid; (**d**) ultrasonic time versus particle size; (**e**) ultrasonic time versus ratio of liquid to solid; (**f**) particle size versus ratio of liquid to solid.

**Figure 3:**
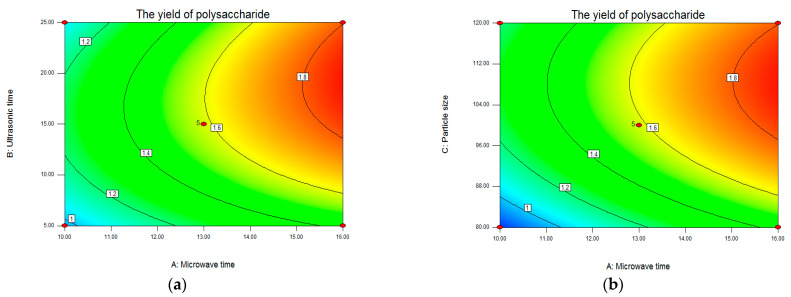

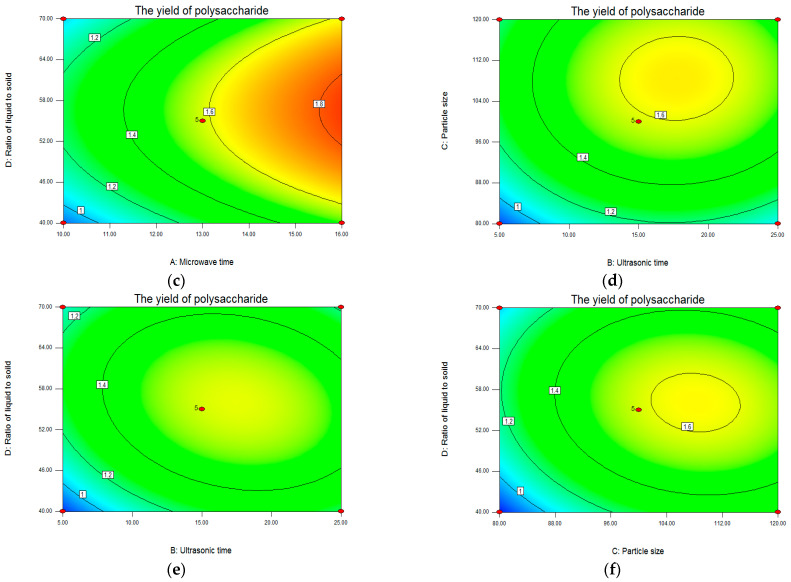
Contour plots of ultrasound–microwave combined extraction. (**a**) microwave time versus ultrasonic time; (**b**) microwave time versus particle size; (**c**) microwave time versus ratio of liquid to solid; (**d**) ultrasonic time versus particle size; (**e**) ultrasonic time versus ratio of liquid to solid; (**f**) particle size versus ratio of liquid to solid.

The synergetic effect of the selected four factors on polysaccharide yield was shown in Figure 2 (3D graphs) and Figure 3 (contour plots). The two factors were fixed at 0 levels, and the impact of the other two factors on the response value was analyzed. Figure 2a–c showed the interactions of microwave time and ultrasonic time, microwave time and particle size, and microwave time and ratio of liquid–solid on LLP yield, respectively. A three-dimensional graph (Figure 2a) and contour plot (Figure 3a) showed that the interaction of microwave time and ultrasonic time on the yield when particle size and ratio of liquid to solid were designated as intermediate values. In Figure 2a, blue and red areas represented low and high yields of LLP, respectively. The yield increased with the prolongation of microwave time, while the yield increased first and then declined slightly with the prolongation of ultrasonic time. In Figure 3a, the more intensive contours meant the greater impact on the yield. The contour lines of microwave time were denser than those of ultrasonic time, indicating that the effect of microwave time on the yield was stronger than that of ultrasonic time. Based on response surface analysis, it is speculated that the maximum yield (1.90%) is accessible when microwave time and ultrasonic time are 16 min and 25 min, respectively.

Figure 2d,e, respectively, represented the interaction of ultrasonic time and particle size and the interaction of ultrasonic time and liquid–solid ratio. The 3D graph (Figure 2e) and contour plot (Figure 3e) showed that the interaction of ultrasonic time and ratio of liquid to solid when microwave time and particle size were set at the intermediate levels. With the prolongation of ultrasonic time, the yield increased first and then decreased (Figure 2e). With the increase of ratio of liquid to solid, the yield also increased first and then declined (Figure 2e). Thus, there was the maximum point at the center of the pattern. The shape of the contours represents the interaction of each factor on the response value. Among them, the ellipse represents a strong influence (*p* < 0.05), while the circle represents a weak influence (*p* > 0.05). The interaction between ultrasonic time and ratio of liquid to solid (appeared in the ellipse in Figure 3e) had a stronger influence on the yield than the interaction between ultrasonic time and particle size (appeared in the circle in Figure 3d). In Figure 2f and Figure 3f, few red areas were observed, suggesting that the synergetic effect of particle size and liquid–solid ratio on the yield was insignificant. The findings in Figure 2 and Figure 3 are in accordance with the results of ANOVA in Table 3, indicating that the model established in this study is suitable to predict the optimal conditions for LLP extraction.

According to the 3D response surface plot, contour plot, and regression analysis, the interactive effects of each factor on the response value were insignificant (*p* > 0.05). Hence, the influence of microwave time, ultrasonic time, particle size, and liquid–solid ratio on LLP yield was independent. Based on the experimental data and parameter correction, the optimal conditions for UMCE of LLP were as follows: microwave time of 15.99 min, ultrasonic time of 19.94 min, particle size of 105.71 mesh, and ratio of liquid to solid of 55.55:1. Under the deduced conditions, the yield of LLP was predicted to be 1.905%. Taking into account the practical operability, the optimum extraction conditions were modified as follows: microwave time of 16 min, ultrasonic time of 20 min, particle size of 100 mesh, and liquid–solid ratio of 55:1.

#### 2.1.3. Verification of Ultrasound–Microwave Combined Extraction

The availability of the established RSM model for predicting the optimum UMCE parameters was tested under the deduced conditions (microwave time of 16 min, ultrasonic time of 20 min, particle size of 100 mesh, and liquid–solid ratio of 55:1). Additionally, the relative high yield (1.873 ± 0.001%) was achieved, demonstrating that the RSM model was suitable for the optimization of UMCE parameters. Extraction yield of *Lycium barbarum* leaf polysaccharides by UMCE (1.873 ± 0.001%) was significantly higher than those by HWE (1.509 ± 0.004%), UAE (1.182 ± 0.010%), and MAE (0.891 ± 0.050%) (*p* < 0.05).

### 2.2. Chemical Characterization of Polysaccharides and Their Fractions

In order to understand chemical and physical properties of LLP samples, biochemical assays (including Molish assay, iodine–potassium iodide assay, and Fehling’s reaction) were performed, and ultraviolet spectrum, circular dichroism, and high-performance liquid chromatograms (monosaccharide composition) as well as scanning electron micrographs of LLP samples were analyzed (Figures S1–S4). The results of Molish reaction showed that there was a purple ring at the surface of the solution of LLP samples and concentrated sulfuric acid, indicating that the main constituent of the samples was carbohydrates. When Fehling’s reagent was added into the solution of LLP samples, there was no brick-red precipitate. The results of Fehling’s reaction confirmed that the samples did not contain reducing sugar. In the iodine–potassium iodide test, the reaction solution did not translate into blue when iodine–potassium iodide solution was added, indicating that the samples did not contain amylose and/or amylopectin.

### 2.3. In Vitro Hypoglycemic and Antioxidant Activities of LLP

#### 2.3.1. Antioxidant Activity

**Figure 4:**
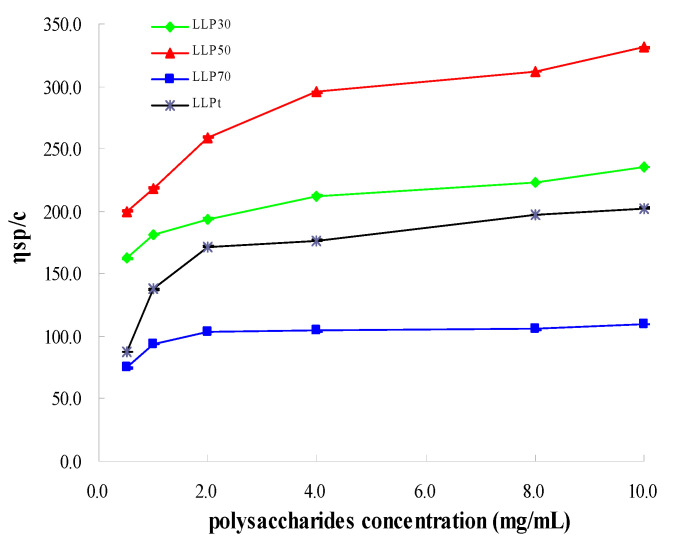
Viscosity-average molecular weights of LLP.

**Figure 5:**
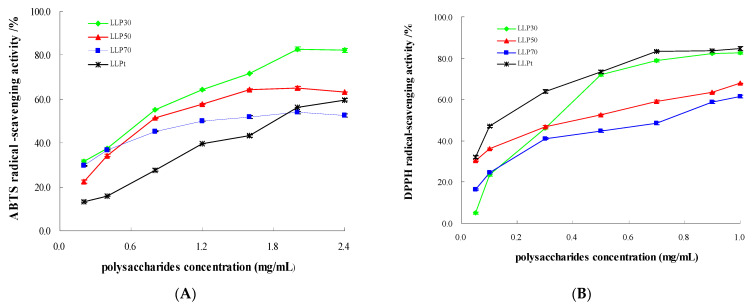
Antioxidant activities of polysaccharides extracted from *Lycium barbarum* leaves. (**A**) ABTS assay; (**B**) DPPH assay.

To elucidate bioactivities of polysaccharides extracted from *Lycium barbarum* leaves, total polysaccharides (LLP_t_) were fractioned according to their molecular weights by gradient ethanol precipitation method, and the resulting fractions with various molecular weights were, respectively, assigned as LLP_30_, LLP_50_, and LLP_70_ (Figure 4). Subsequently, hypoglycemic and antioxidant activities of LLP_30_, LLP_50_, LLP_70_, and LLP_t_ were analyzed. The viscosity-average molecular weights of LLP_30_, LLP_50_, and LLP_70_ were 8.0×10^4^, 9.8 × 10^4^, and 4.4 × 10^4^, respectively. As shown in Figure 5A, scavenging activities against ABTS radicals were dose-dependent, and the maximum scavenging rate exceeded 80%. Scavenging ability against ABTS radicals decreased in the order: LLP_30_ > LLP_50_ > LLP_70_ > LLP_t_ (Table 4). All of three novel fractions (LLP_30_, LLP_50_, and LLP_70_) exhibited higher antioxidant activity against ABTS radicals than total polysaccharides (LLP_t_) (*p* < 0.05). As shown in Figure 5B, polysaccharides from *Lycium barbarum* leaves displayed a dose-dependent scavenging activity against DPPH radicals, although their activities were lower than that of ascorbic acid (Vc). The scavenging ability against DPPH radicals of LLP_30_ was stronger than those of LLP_50_ and LLP_70_ (*p* < 0.05), which was in accord with the tendency in ABTS assay (Table 4). To sum up, antioxidant activity of LLP_30_ was higher than other two fractions (LLP_50_ and LLP_70_), suggesting that polysaccharide fractions with various molecular weights possessed different antioxidant ability.

#### 2.3.2. Inhibitory Effect on α-Glucosidase

**Figure 6:**
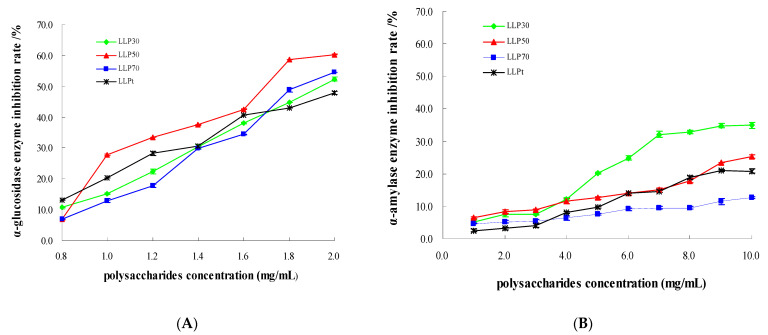
In vitro hypoglycemic activities of polysaccharides extracted from *Lycium barbarum* leaves. (**A**) α-glucosidase; (**B**) α-amylase.

An inhibitory effect of polysaccharides extracted from *Lycium barbarum* leaves on α-glucosidase was observed in an obvious dose-dependent pattern in the range of 0.8~2.0 mg/mL (Figure 6A). All of the polysaccharide samples were able to suppress α-glucosidase activity, although their inhibitory ability was lower than that of acarbose (Table 5). The maximum inhibition rate of polysaccharide samples against α-glucosidase reached 60.3% (Figure 6A). Similarly, polysaccharides extracted from *Nelumbo nucifera* exerted a remarked inhibitory effect on α-glucosidase activity [20]. The correlation coefficient of regression equations between concentrations of polysaccharide samples and inhibition rates ranged from 0.9507 to 0.9962 (Table 5), indicating that these mathematic models well fitted the relationships between concentrations of polysaccharide samples and inhibition rates. The IC_50_ (concentration that inhibited enzyme activity by 50%) of three fractions (1.659~1.945 mg/mL) was lower than that of LLP_t_ (2.101 mg/mL) (*p* > 0.05) (Table 5), implying that inhibitory ability of three fractions was slightly stronger than that of total polysaccharides.

#### 2.3.3. Inhibitory Effect on α-Amylase

Polysaccharides extracted from *Lycium barbarum* leaves notably inhibited α-amylase activity in a dose-dependent manner (Figure 6B). IC_50_ of LLP_t_ was higher than that of LLP_30_ and LLP_50_ (Table 5), suggesting that inhibitory ability of LLP_30_ and LLP_50_ was stronger than that of LLP_t_. Among three fractions, LLP_30_ had the lowest IC_50_ when compared to LLP_50_ and LLP_70_ (Table 5), implying that inhibitory ability of LLP_30_ was stronger than that of LLP_50_ and LLP_70_. The correlation coefficient of regression equations between concentrations of polysaccharide samples and inhibition rates ranged from 0.9469 to 0.9800 (Table 5), indicating that these mathematic models well fitted dose–effect relationships.

## 3. Discussion

An ultrasound–microwave combined extraction procedure of *Lycium barbarum* leaf polysaccharides was developed by response surface method. Compared with traditional extraction methods such as HWE, UAE, and MAE, UMCE achieved the highest LLP yield. The high yield in UMCE may be attributed to the sequential application of ultrasound and microwave. The cavitation caused by ultrasound may destroy cell walls of *Lycium barbarum* leaves, and the heating effect generated after microwave irradiation may accelerate the dissolution of entocytes, both of which lead to the enhanced accessibility of polysaccharides [12,21].

Polysaccharides extracted from *Lycium barbarum* leaves by UMCE were found to possess antioxidant capacities, which were presumably due to their enrichment of hydroxyl groups and aldehyde groups [22]. Numerous papers have reported the correlation between oxidative stress and diabetes [1]. On the one hand, excessive amounts of free radicals might cause diabetes complications. On the other hand, hyperglycemia might induce oxidative stress [23]. Antioxidants might be helpful to the reduction of diabetes occurrence [24]. Consequently, it is of paramount importance to further elucidate in vivo antioxidant ability of polysaccharides from *Lycium barbarum* leaves.

Remarkably, *Lycium barbarum* leaf polysaccharides inhibited α-amylase and α-glucosidase activities. These two enzymes are capable of breaking (α1→4) glycosidic bonds between glucose units. They play prominent roles in the digestion of starch, which is the major source of carbohydrates for most humans [1]. α-amylase and α-glucosidase inhibitors are regarded as an important source of functional food ingredients or phytomedicines for preventing and treating hyperglycemia or diabetes [25]. For instance, acarbose has been developed and marketed due to its inhibitory ability against α-glucosidase and α-amylase [25]. Recently, some side effects of acarbose were found in clinic practice, such as stomachache, meteorism, and diarrhea, which might be related to excessive inhibition of pancreatic α-amylase. By contrast, inhibitory activities against α-amylase and α-glucosidase of LLP were moderate (Table 5), which might allow for the minimization of the above-mentioned side effects. Among three fractions (LLP_30_, LLP_50_, and LLP_70_), LLP_30_ exhibited relatively high hypoglycemic and antioxidant activities in vitro, which showed a potential for becoming a nutraceutical or a phytopharmaceutical for prevention and treatment of hyperglycemia or diabetes.

Generally, bioactivities of polysaccharides are intimately linked with their chemical structure [22]. Polysaccharide samples prepared from *Lycium barbarum* leaves with various molecular weights exhibited different hypoglycemic and antioxidant capacities in vitro. Similarly, a huge variation in antioxidant activity and inhibitory activity against HepG2 cells was observed among polysaccharide samples from alfalfa roots with different molecular weights [1]. To reveal the structure–activity relationship (including the correlation between molecular weights and hypoglycemic and antioxidant activities) of polysaccharide samples from *Lycium barbarum* leaves, further study is needed, in vitro as well as in vivo.

## 4. Materials and Methods

### 4.1. Plant Materials and Reagents

*Lycium barbarum* leaves were purchased from Yinchuan YX Goji Co., Ltd. (Yinchuan, China) and were authenticated by one of the authors (H.F.Z.). The voucher specimens were deposited at International Joint Research Center of Shaanxi Province for Food and Health Sciences (Xi’an, China) for future references. ABTS (2,2′-azino-bis-(3-ethyl-benzthiazoline-6-sulfonic acid) diammonium salt) and DPPH (1,1-diphenyl-2-picrylhydrazyl) were bought from Sigma Co., Ltd. (St. Louis, MO, USA). α-glucosidase (50 U/mg) and α-_D_-glucopyranoside (PNPG) were purchased from YY Biotechnology Co., Ltd. (Shanghai, China). α-amylase (3.7 U/mg) was provided by Aoboxing Bio-tech Co., Ltd. (Beijing, China). Ascorbic acid (purity ≥ 99%) was bought from Sinopharm Chemical Reagent Co., Ltd. (Shanghai, China). Acarbose was purchased from Bayer AG (Werk Leverkusen, Germany). Other chemical reagents were of analytical grade.

### 4.2. Polysaccharides Extraction

#### 4.2.1. UMCE Method

*Lycium barbarum* leaves were pulverized and then sieved through different-sized screens (10, 40, 60, 80, 100, and 120 mesh). The milled leaf powders of 1.5 g were added into a certain volume of distilled water. The mixture was placed in a CW-2000 microwave-ultrasound synergistic extraction apparatus (XTrust Instrument Co., Ltd., Shanghai, China) for a specific time [15]. Afterward, the processed mixture was centrifuged at 4000 rpm for 10 min, and the supernatant was concentrated in a RE-52AA rotary evaporator (Shanghai YR Co., Ltd., Shanghai, China) under reduced pressure. Then, four times the volume of absolute ethanol was added to the concentrated solution to precipitate polysaccharides at 4 °C for 12 h. Finally, the precipitate was separated by centrifugation at 4200 rpm for 15 min and stored as crude polysaccharides at 4 °C for further use.

#### 4.2.2. Conventional Extraction Methods

Hot water extraction, microwave-assisted extraction, and ultrasonic-assisted extraction were conducted as previously described [15,22]. With the exception of extracting temperature, other parameters (extraction time, particle size, and ratio of liquid to solid) were consistent with the optimized conditions of UMCE.

#### 4.2.3. Determination of Extraction Yield of Polysaccharides

Contents of polysaccharides were quantified according to the phenol-sulfuric acid method optimized in our laboratory [26]. The regression equation of calibration curve based upon the linear relationship between concentration of carbohydrate solution (*X*) and absorbance at 490 nm (*Y*) was established as follows: *Y* = 9.0606*X* − 0.0073 (*R*^2^ = 0.9960). Extraction yield of polysaccharides from *Lycium barbarum* leaves was calculated according to the following equation:

Y = *m*/*M* × 100

where Y represented extraction yield of polysaccharides (%); *m* represented mass (g) of polysaccharides extracted; and *M* represented mass (g) of leaf powders.

### 4.3. Purification and Fractionation of Polysaccharides

Crude polysaccharides were purified according to previously reported protocols [27,28]. The purified samples were lyophilized using a FDU-1200 freeze drier (Tokyo Rikakikai Co., Ltd., Tokyo, Japan), and the dried polysaccharides were named total polysaccharides (LLPt). LLPt was further fractionated according to a previously reported protocol of gradient ethanol precipitation in our laboratory [28] with some modifications. In brief, aqueous ethanol solutions at varying concentrations (30%, 50%, and 70%, *v*/*v*) were applied to fractionation of LLPt, and then three novel polysaccharide fractions with different molecular weights were achieved. The purified fractions were labeled LLP_30_, LLP_50_, and LLP_70_, respectively.

### 4.4. Qualitative Analysis of Polysaccharides

#### 4.4.1. Molish Assay

Molish reaction of polysaccharide samples from *Lycium barbarum* leaves was conducted as previously described in our laboratory [22]. Instead of polysaccharide samples solution, distilled water and glucose solution were used as the negative and positive controls, respectively.

#### 4.4.2. Fehling’s Reaction

Fehling’s reaction of polysaccharide samples was performed using the method reported in our laboratory [28] with slight modifications. Briefly, polysaccharide solution (1 mg/mL) of 2 mL was added to a tube, followed by the addition of Fehling’s reagent of 1 mL. Then, the tube was incubated at 60 °C for 2 min to observe the color change. Distilled water and glucose solution were used as the negative and positive controls, respectively.

#### 4.4.3. Iodine–Potassium Iodide Method

Iodine–potassium iodide assay was carried out according to a previously reported protocol [28]. Distilled water and starch solution were used as the negative and positive controls, respectively.

### 4.5. Measurement of Viscosity-Average Molecular Weights

Molecular weights of polysaccharide samples were determined using a Ubbelohde viscometer (capillary diameter = 0.55 mm) according to the method described by Ma et al. (2021) [29]. Molecular weight was calculated according to Mark-Houwink equation:

[*η*] = *K* × *M*_w_^α^

where [*η*] represented intrinsic viscosity; *K* was a constant (7 × 10^4^); α was an exponent (1.10); and *M*_w_ represented molecular weights.

### 4.6. Antioxidant Activities of LLP In Vitro

#### 4.6.1. Scavenging Ability of LLP on ABTS Radicals

Scavenging activities of LLP on ABTS radicals were determined according to a previously reported protocol [30] with slight modification. At first, ABTS solutions of 4.75 mL were added to sample solutions of 0.25 mL at different concentrations (0.20, 0.40, 0.80, 1.20, 1.60, 2.00, and 2.40 mg/mL). Then, the mixtures were incubated at 30 °C in dark for 6 min, and absorbances were measured at the wavelength of 734 nm. EC_50_ (concentration that scavenged free radicals by 50%) of LLP was calculated as described previously [22]. Distilled water and ascorbic acid solution were used as the negative and positive controls, respectively.

#### 4.6.2. Scavenging Ability of LLP on DPPH Radicals

Assay of scavenging ability of LLP on DPPH radicals was modified from Yang et al. (2017) [22]. Briefly, DPPH solutions (0.2 mmol/L) of 1.0 mL were added to sample solutions of 2.0 mL at various concentrations (0.05, 0.10, 0.30, 0.50, 0.70, 0.90, and 1.00 mg/mL). The resulting mixtures were incubated at 37 °C for 30 min, and absorbances were determined at 517 nm. EC_50_ of LLP was calculated according to Yang et al. (2017) [22]. Distilled water and ascorbic acid solution were used as the negative and positive controls, respectively.

### 4.7. Hypoglycemic Activities of LLP In Vitro

#### 4.7.1. Inhibitory Effects of LLP on α-Glucosidase

Inhibitory activity of LLP against α-glucosidase was measured using the method reported by Wu et al. (2022) [20]. Briefly, PBS buffers (physiological buffered saline, pH = 6.8) of 50 μL and α-glucosidase solutions of 50 μL were added to sample solutions of 50 μL varying in concentrations (0.80, 1.00, 1.20, 1.40, 1.60, and 2.00 mg/mL). The mixtures were incubated at 37 °C for 15 min, followed by the addition of PNPG solutions of 100 μL. Additionally, the resulting mixtures were incubated for 5 min. At last, Na_2_CO_3_ solutions (0.1 mol/L) of 750 μL were added to terminate the reaction. Absorbances were measured at 405 nm, and IC_50_ of LLP was calculated according to the method described by Zhang et al. (2011a) and Wu et al. (2022) [5,20]. PBS buffer and acarbose solution were used as the negative and positive controls, respectively.

#### 4.7.2. Inhibitory Effects of LLP on α-Amylase

Inhibitory activity of LLP against α-amylase was analyzed according to the method developed in our laboratory [31]. In brief, PBS solutions of 50 μL containing soluble starch (10.0 mg/mL) were preheated at 40 °C for 5.0 min. Afterward, sample solutions of 50 μL at various concentrations (1.0, 2.0, 3.0, 4.0, 5.0, 6.0, 7.0, 8.0, 9.0, and 10.0 mg/mL) and α-amylase solutions of 10 μL were sequentially added. After the mixtures were incubated at 40 °C for 7.5 min, iodine solutions (1.3 mmol/L) of 300 μL were added. Then, absorbances were determined at 660 nm. IC_50_ of LLP was calculated using the method reported by An et al. (2020) [31]. PBS buffer and acarbose solution were used as the negative and positive controls, respectively [25].

### 4.8. Experimental Design and Statistical Analysis

#### 4.8.1. BBD

A four-factor, three-level BBD was employed to optimize the conditions for UMCE of polysaccharides from *Lycium barbarum* leaves. Polysaccharide yield was taken as the response value. High, moderate, and low levels of each variable were denoted (1), (0), and (−1), respectively (Table 1). A total of 29 experiments were performed, each of which included three replicates at the center points to evaluate the error. The levels of variables and values of runs are listed in Table 2. The 3D response surface plots and contour plots were used to illustrate the relationship between two independent variables.

#### 4.8.2. Statistical Analysis

All the experiments were repeated at least three times. ANOVA was conducted using DPS 7.5 software (Hangzhou Ruifeng Information Technology Co., Ltd., Hangzhou, China). Additionally, RSM analysis was implemented using Design-Expert 8.0.6 software (Stat-Ease Inc., Minneapolis, MN, USA).

## 5. Conclusions

An ultrasound–microwave combined extraction procedure of polysaccharides from *Lycium barbarum* leaves was developed by response surface method. UMCE showed the highest LLP yield in comparison with conventional extraction methods such as HWE, UAE, and MAE. Polysaccharide samples extracted by UMCE possessed scavenging capacities against ABTS and DPPH radicals, as well as inhibitory activities against α-glucosidase and α-amylase. Among three novel polysaccharide fractions with different molecular weights obtained by gradient ethanol precipitation, LLP_30_ exhibited relatively high antioxidant and hypoglycemic activities in vitro, which showed a potential for becoming a nutraceutical or a phytopharmaceutical for prevention and treatment of hyperglycemia or diabetes. The present research provides clues on the utility of *Lycium barbarum* leaves as a source of natural antioxidants and anti-diabetic phytochemicals.

## Figures and Tables

**Table 1:** Coded values of independent variables.

Variables	Symbol Codes	Levels		
		1	0	−1
Microwave time (min)	A	10	13	16
Ultrasonic time (min)	B	5	15	25
Particle size (mesh)	C	80	100	120
Ratio of liquid to solid (mL/g)	D	40	55	70

**Table 2:** Levels of independent variables and Box–Behnken Design arrangement.

Runs	Microwave Time A (min)	Ultrasonic Time B (min)	Particle Size C (mesh)	Ratio of Liquid to Solid D (mL/g)	Polysaccharide Yield Y (%)
1	−1 (10)	0 (15)	1 (120)	0 (55)	1.184 ± 0.171
2	0 (13)	0 (15)	0 (100)	0 (55)	1.589 ± 0.090
3	−1 (10)	0 (15)	−1 (80)	0 (55)	0.902 ± 0.132
4	−1 (10)	0 (15)	0 (100)	1 (70)	1.022 ± 0.240
5	0 (13)	0 (15)	0 (100)	0 (55)	1.557 ± 0.046
6	0 (13)	−1 (5)	0 (100)	−1 (40)	0.930 ± 0.223
7	1 (16)	0 (15)	1 (120)	0 (55)	1.763 ± 0.139
8	0 (13)	−1 (5)	0 (100)	1 (70)	1.242 ± 0.070
9	−1 (10)	−1 (5)	0 (100)	0 (55)	0.869 ± 0.051
10	0 (13)	1 (25)	1 (120)	0 (55)	1.439 ± 0.126
11	1 (16)	0 (15)	0 (100)	−1 (40)	1.456 ± 0.274
12	0 (13)	0 (15)	−1 (80)	1 (70)	0.931 ± 0.089
13	0 (13)	1 (25)	−1 (80)	0 (55)	1.082 ± 0.210
14	0 (13)	0 (15)	0 (100)	0 (55)	1.521 ± 0.032
15	−1 (10)	0 (15)	0 (100)	−1 (40)	0.889 ± 0.110
16	0 (13)	1 (25)	0 (100)	−1 (40)	1.131 ± 0.135
17	0 (13)	0 (15)	0 (100)	0 (55)	1.627 ± 0.239
18	0 (13)	0 (15)	−1 (80)	−1 (40)	0.775 ± 0.101
19	0 (13)	0 (15)	1 (120)	1 (70)	1.317 ± 0.183
20	1 (16)	−1 (5)	0 (100)	0 (55)	1.372 ± 0.063
21	−1 (10)	1 (25)	0 (100)	0 (55)	1.103 ± 0.211
22	0 (13)	1 (25)	0 (100)	1 (70)	1.114 ± 0.088
23	0 (13)	−1 (5)	−1 (80)	0 (55)	0.839 ± 0.197
24	0 (13)	0 (15)	1 (120)	−1 (40)	1.338 ± 0.142
25	1 (16)	0 (15)	0 (100)	1 (70)	1.596 ± 0.111
26	0 (13)	0 (15)	0 (100)	0 (55)	1.621 ± 0.229
27	1 (16)	0 (15)	−1 (80)	0 (55)	1.468 ± 0.243
28	1 (16)	1 (25)	0 (100)	0 (55)	1.898 ± 0.215
29	0 (13)	−1 (5)	1 (120)	0 (55)	1.142 ± 0.153

Note: Results of polysaccharide yields were expressed as mean ± SD (standard deviation).

**Table 3:** Analysis of variance for fitted regression model.

Source	Sum of Squares	df	Mean Square	F-Value	*p*-Value	Significance
Model	2.555	14	0.183	25.344	<0.0001	**
A	1.070	1	1.070	148.624	<0.0001	**
B	0.157	1	0.157	21.812	0.0004	**
C	0.398	1	0.398	55.291	<0.0001	**
D	0.041	1	0.041	5.718	0.0314	*
AB	0.021	1	0.021	2.960	0.1074	
AC	0.000	1	0.000	0.006	0.9400	
AD	0.000	1	0.000	0.002	0.9677	
BC	0.001	1	0.001	0.101	0.7551	
BD	0.027	1	0.027	3.757	0.0730	
CD	0.008	1	0.008	1.088	0.3147	
A^2^	0.017	1	0.017	2.373	0.1457	
B^2^	0.318	1	0.318	44.170	<0.0001	**
C^2^	0.311	1	0.311	43.228	<0.0001	**
D^2^	0.487	1	0.487	67.594	<0.0001	**
Residual	0.101	14	0.007			
Lack of fit	0.093	10	0.009	4.682	0.0751	
Pure Error	0.008	4	0.002			
Total	2.656	28				

Note: *, significant (*p* < 0.05); ** very significant (*p* < 0.01).

**Table 4:** Fitting equations of dose-effect relationships between radical-scavenging capacities and mass concentrations of polysaccharides extracted from *Lycium barbarum* leaves and their EC_50_.

Samples	ABTS			DPPH		
	Fitting Equations	*R* ^2^	EC_50_ (mg/mL)	Fitting Equations	*R* ^2^	EC_50_ (mg/mL)
V_C_	Y = −949942X^2^ + 18852X + 7.6294	0.9916	0.003	Y = 27.688 ln(X) + 231.41	0.9625	0.002
LLP_t_	Y = 22.243X + 9.2361	0.9809	1.833	Y = 17.423 ln(X) + 85.569	0.9877	0.129
LLP_30_	Y = −9.399X^2^ + 48.731X + 20.263	0.9870	0.706	Y = 16.775 ln(X) + 80.264	0.9784	0.165
LLP_50_	Y = −13.29X^2^ + 50.981X + 16.908	0.9899	0.828	Y = −21.895X^2^ + 59.008X + 29.253	0.9889	0.416
LLP_70_	Y = −6.6817X^2^ + 26.332X + 27.829	0.9920	1.219	Y = 14.259 ln(X) + 57.793	0.9697	0.579

Note: X and Y represented LLP concentrations (mg/mL) and radical-scavenging rates (%), respectively.

**Table 5:** Fitting equations of dose–effect relationships between enzyme inhibition rates and mass concentrations of polysaccharides extracted from *Lycium barbarum* leaves and their IC_50_.

Samples	α-Glucosidase			α-Amylase		
	Fitting Equations	*R* ^2^	IC_50_ (mg/mL)	Fitting Equations	*R* ^2^	IC_50_ (mg/mL)
Acarbose	Y = 14.131 ln(X) + 70.565	0.9534	0.0002	Y = 3.046X^2^ − 7.8893X + 8.9804	0.9960	5.187
LLP_t_	Y = −7.225X^2^ + 49.279X − 21.649	0.9871	2.101	Y = 2.3427X − 1.2074	0.9744	21.858
LLP_30_	Y = 35.678X − 19.384	0.9962	1.945	Y = −0.1034X^2^ + 5.076X − 2.7315	0.9469	14.928
LLP_50_	Y = 54.854 ln(X) + 22.13	0.9507	1.659	Y = 0.1567X^2^ + 0.2861X + 6.7586	0.9800	15.721
LLP_70_	Y = 41.227X − 28.403	0.9813	1.902	Y = 0.0343X^2^ + 0.5153X + 3.9659	0.9760	29.885

Note: X and Y represented LLP concentrations (mg/mL) and enzyme inhibition rates (%), respectively.

## Data Availability

Not applicable.

## Supplementary Materials

The following supporting information can be downloaded at: https://www.mdpi.com/article/10.3390/molecules28093880/s1. Figure S1: Ultraviolet spectrum of LLP; Figure S2: Circular dichroism of LLP; Figure S3: High-performance liquid chromatograms of standard monosaccharides (A) and LLP (B); Figure S4: Scanning electron micrographs of LLP.

## References

[1] Duan W.X., Yang X.H., Zhang H.F., Feng J., Zhang M.Y. (2022). Chemical structure, hypoglycemic activity and mechanism of action of selenium-polysaccharides. Biol. Trace Elem. Res..

[2] Xu X., Shan B., Liao C.H., Xie J.H., Wen P.W., Shid J.Y. (2015). Anti-diabetic properties of *Momordica charantia* L. polysaccharide inalloxan-induced diabetic mice. Int. J. Biol. Macromol..

[3] Zaghloul N., Awaisu A., Mahfouz A., Alyafei S., Elewa H. (2022). A 5-year trend in the use of sodium-glucose co-transporter 2 inhibitors and other oral antidiabetic drugs in a Middle Eastern country. Int. J. Clin. Pharm..

[4] Lee T.T.L., Hui J.M.H., Lee Y.H.A., Satti D.I., Shum Y.K.L., Kiu P.T.H., Wai A.K.C., Liu T., Wong W.T., Chan J.S.K. (2022). Sulfonylurea is associated with higher risks of ventricular arrhythmia or sudden cardiac death compared with metformin: A population-based Cohort study. J. Am. Heart Assoc..

[5] Zhang L., Hogan S., Li J.R., Sun S., Canning C., Zheng S.J., Zhou K.Q. (2011). Grape skin extract inhibits mammalian intestinal α-glucosidase activity and suppresses postprandial glycemic response in streptozonic-treated mice. Food Chem..

[6] Zhang H.F., Yang X.H. (2010). Bioactive substances in *Lycium* leaves and their application to food industry. Sci. Technol. Food Ind..

[7] Tang H.L., Chen C., Wang S.K., Sun G.J. (2015). Biochemical analysis and hypoglycemic activity of a polysaccharide isolated from the fruit of *Lycium barbarum* L.. Int. J. Biol. Macromol..

[8] Qi Y., Duan G., Fan G., Peng N. (2022). Effect of *Lycium barbarum* polysaccharides on cell signal transduction pathways. Biomed. Pharmacother..

[9] Dong J.Z., Lu D.Y., Wang Y. (2009). Analysis of flavonoids from leaves of cultivated *Lycium barbarum* L.. Plant Foods Hum. Nutr..

[10] Quan N., Wang Q., Li X.Y., Yang X.H., Zhu C.Y., Zhang H.F. (2018). Antioxidant capacity and inhibitory activity against DNA damage of *Lycium barbarum* leaves and fruits. Nat. Prod. Res. Dev..

[11] Jiang L., Mei L.J., Liu Z.G., Li J.Q., Wang Q.L., Shao Y., Tao Y.D. (2013). Extraction and hypoglycemic effect of crude polysaccharides from Chinese wolfberry (*Lycium chinense*) leaves. Food Sci..

[12] Zhang H.F., Yang X.H., Wang Y. (2011). Microwave assisted extraction of secondary metabolites from plants: Current status and future directions. Trends Food Sci. Technol..

[13] Khedmat L., Izadi A., Mofid V., Mojtahedi S.Y. (2020). Recent advances in extracting pectin by single and combined ultrasound techniques: A review of techno-functional and bioactive health-promoting aspects. Carbohydr. Polym..

[14] Zeng S., Wang B., Lv W., Wu Y. (2023). Effects of microwave power and hot air temperature on the physicochemical properties of dried ginger (*Zingiber officinale*) using microwave hot-air rolling drying. Food Chem..

[15] Zeng H.L., Zhang Y., Lin S., Jian Y.Y., Miao S., Zheng B.D. (2015). Ultrasonic-microwave synergistic extraction (UMSE) and molecular weight distribution of polysaccharides from *Fortunella margarita* (Lour.) swingle. Sep. Purif. Technol..

[16] Michalaki A., Karantonis H.C., Kritikou A.S., Thomaidis N.S., Dasenaki M.E. (2023). Ultrasound-assisted extraction of total phenolic compounds and antioxidant activity evaluation from Oregano (*Origanum vulgare* ssp. *hirtum*) using response surface methodology and identification of specific phenolic compounds with HPLC-PDA and Q-TOF-MS/MS. Molecules.

[17] Prabhu A.A., Mandal B., Dasu V.V. (2017). Medium optimization for high yield production of extracellular human interferon-γ from *Pichia pastoris*: A statistical optimizationand neural network-based approach. Korean J. Chem. Eng..

[18] Zhao G., Wang H., Liu G., Wang Z.Q. (2016). Box-Behnken response surface design for the optimization of electrochemical detection of cadmium by square wave anodic stripping voltammetry on bismuth film/glassy carbon electrode. Sens. Actuators B Chem..

[19] Liu Y., Qiang M.L., Sun Z.G., Du Y.Q. (2015). Optimization of ultrasonic extraction of polysaccharides from *Hovenia dulcis* peduncles and their antioxidant potential. Int. J. Biol. Macromol..

[20] Wu H., Shu L., Liang T., Li Y., Liu Y., Zhong X., Xing L., Zeng W., Zhao R., Wang X. (2022). Extraction optimization, physicochemical property, antioxidant activity, and α-glucosidase inhibitory effect of polysaccharides from lotus seedpods. J. Sci. Food Agric..

[21] Zhang Y., Lei Y., Qi S., Fan M., Zheng S., Huang Q., Lu X. (2023). Ultrasonic-microwave-assisted extraction for enhancing antioxidant activity of *Dictyophora indusiata* polysaccharides: The difference mechanisms between single and combined assisted extraction. Ultrason. Sonochem..

[22] Yang J., Zhang H.F., Cao X.Y., Yang X.H., Wang F.Z., Guo Q., Sun C.Q. (2017). Enzymatic water extraction of polysaccharides from *Epimedium brevicornu* and their antioxidant activity and protective effect against DNA damage. J. Food Biochem..

[23] Yaribeygi H., Atkin S.L., Sahebkar A. (2019). A review of the molecular mechanisms of hyperglycemia-induced free radical generation leading to oxidative stress. J. Cell. Physiol..

[24] Neha K., Haider M.R., Pathak A., Yar M.S. (2019). Medicinal prospects of antioxidants: A review. Eur. J. Med. Chem..

[25] Sintsova O., Gladkikh I., Kalinovskii A., Zelepuga E., Monastyrnaya M., Kim N., Shevchenko L., Peigneur S., Tytgat J., Kozlovskaya E. (2019). Magnificamide, a β-defensin-like peptide from the mucus of the sea anemone *Heteractis magnifica*, is a strong inhibitor of mammalian α-amylases. Mar. Drugs.

[26] Zhang H.F., Niu L.L., Yang X.H., Li L. (2014). Analysis of water-soluble polysaccharides in an edible medicinal plant *Epimedium*: Method development, validation, and application. J. AOAC Int..

[27] Wei C., He P., He L., Ye X., Cheng J., Wang Y., Li W., Liu Y. (2018). Structure characterization and biological activities of a pectic polysaccharide from cupule of *Castanea henryi*. Int. J. Biol. Macromol..

[28] Zhang M.Y., Yang X.H., Duan M.Y., Zhang H.F. (2022). Structural characterization of novel fraction EP80 of *Epimedium sagittatum* polysaccharides and its effects on enzymes related to alcohol metabolism. Nat. Prod. Res. Dev..

[29] Ma C., Bai J., Shao C., Liu J., Zhang Y., Li X., Yang Y., Xu Y., Wang L. (2021). Degradation of blue honeysuckle polysaccharides, structural characteristics and antiglycation and hypoglycemic activities of degraded products. Food Res. Int..

[30] Zhang H.F., Zhang X., Yang X.F., Qiu N.X., Wang Y., Wang Z.Z. (2013). Microwave assisted extraction of flavonoids from cultivated *Epimedium sagittatum*: Extraction yield and mechanism, antioxidant activity and chemical composition. Ind. Crops Prod..

[31] An Y.J., Zhou X.X., Wang Y., Yang M., Zhang H.F. (2020). Microwave-ultrasound assisted extraction of epimedin F from Epimedii Folium and its effects on α-glucosidase and α-amylase. J. Shaanxi Norm. Univ..

