# Supplementary material for: Ultrasound–Microwave Combined Extraction of Novel Polysaccharide Fractions from Lycium barbarum Leaves and Their In Vitro Hypoglycemic and Antioxidant Activities
Source: Molecules. 2023 May 4;28(9):3880. doi: 10.3390/molecules28093880 (PMC10180117; doi:10.3390/molecules28093880)
Supplement: Supplementary file 1 [file molecules-28-03880-s001.zip › molecules-2341983-supplementary.pdf]

SUPPLEMENTARY MATERIALS

(Additional file for review but not for publication)

FIGURE CAPTIONS

Figure S1. Ultraviolet spectrum of LLP.

Y-coordinate and abscissa represent absorbance and wavelength (nm), respectively.

Figure S2. Circular dichroism of LLP.

Figure S3. High-performance liquid chromatograms of standard monosaccharides (A) and LLP (B).

Peaks: 1, mannose; 2, rhamnose; 3, galacturonic acid; 4, glucose; 5, galactose; 6, arabinose.

Figure S4. Scanning electron micrographs of LLP.

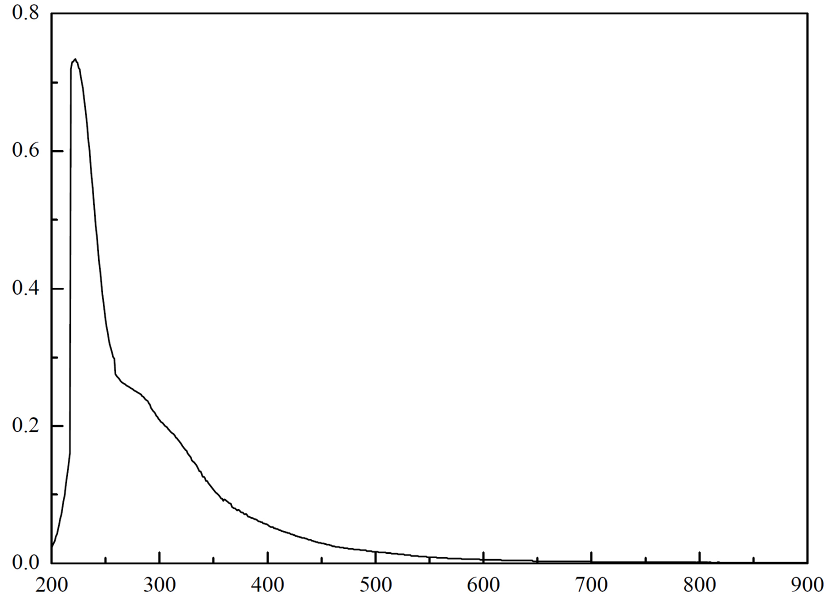

Fig. S1

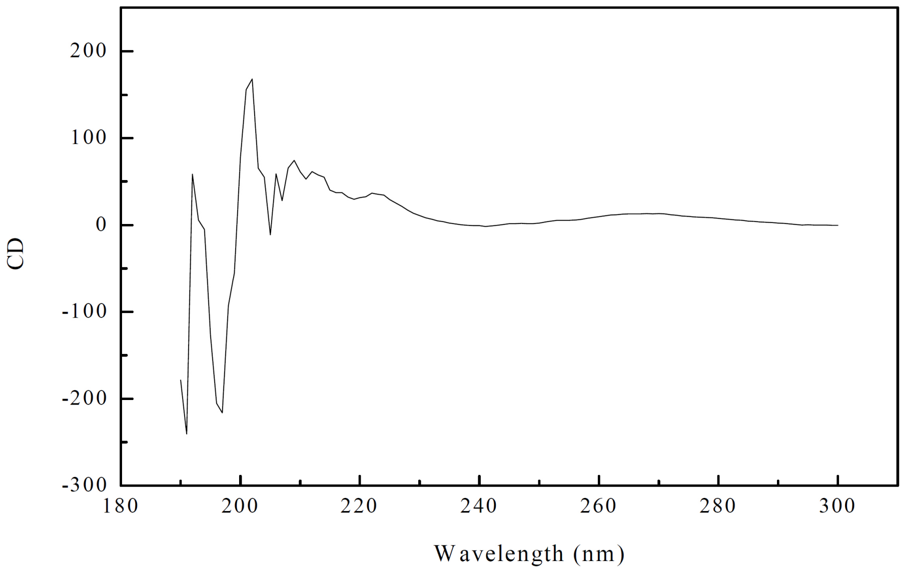
200
100
0
-100
-200
-300
CD
180
200
220
240
260
280
300
Wavelength (nm)

Fig. S2

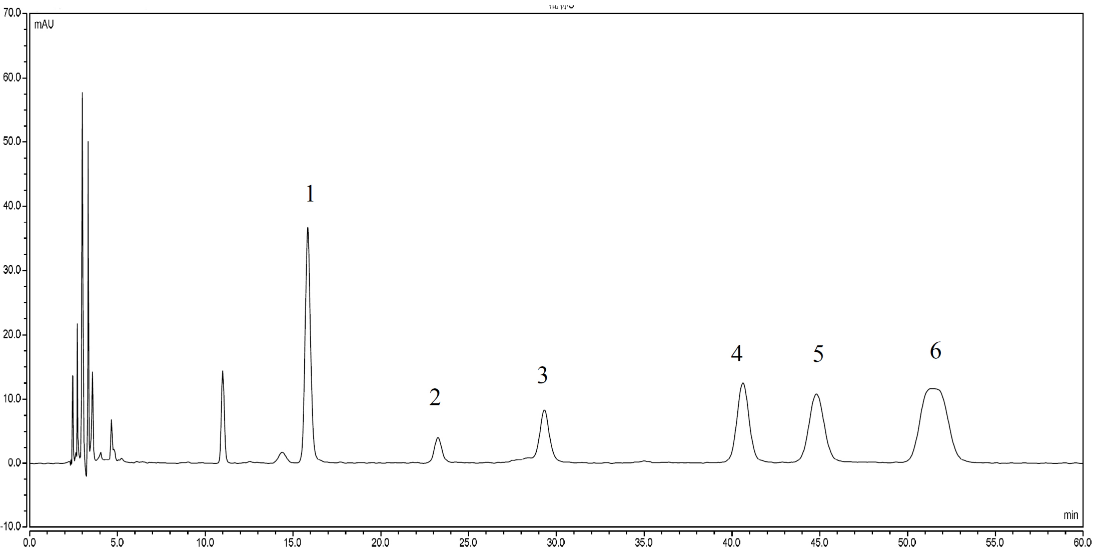
mAU
min
70.0
60.0
50.0
40.0
30.0
20.0
10.0
0.0
-10.0
0.0
5.0
10.0
15.0
20.0
25.0
30.0
35.0
40.0
45.0
50.0
55.0
60.0
1
2
3
4
5
6

A

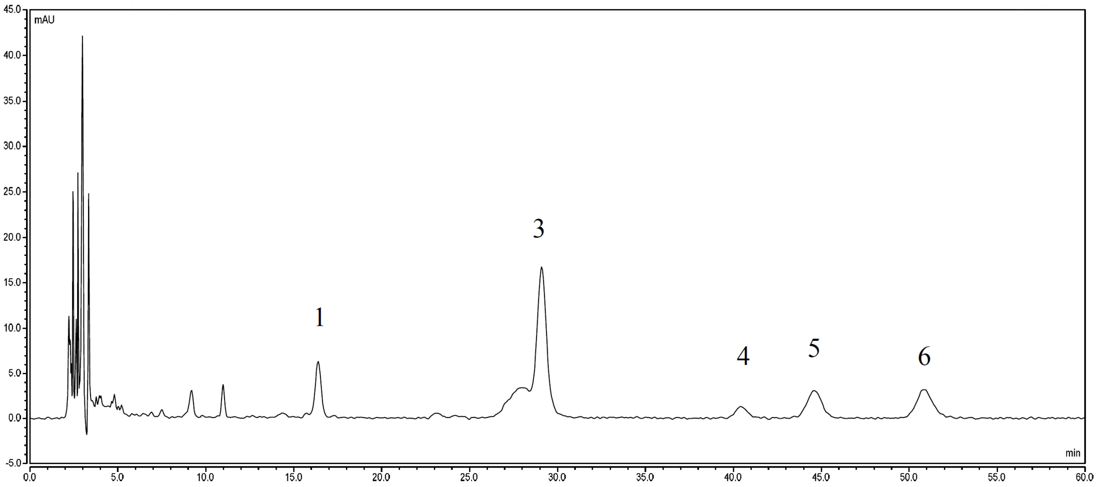
mAU
min
45.0
40.0
35.0
30.0
25.0
20.0
15.0
10.0
5.0
0.0
-5.0
0.0
5.0
10.0
15.0
20.0
25.0
30.0
35.0
40.0
45.0
50.0
55.0
60.0
1
3
4
5
6

B

Fig. S3

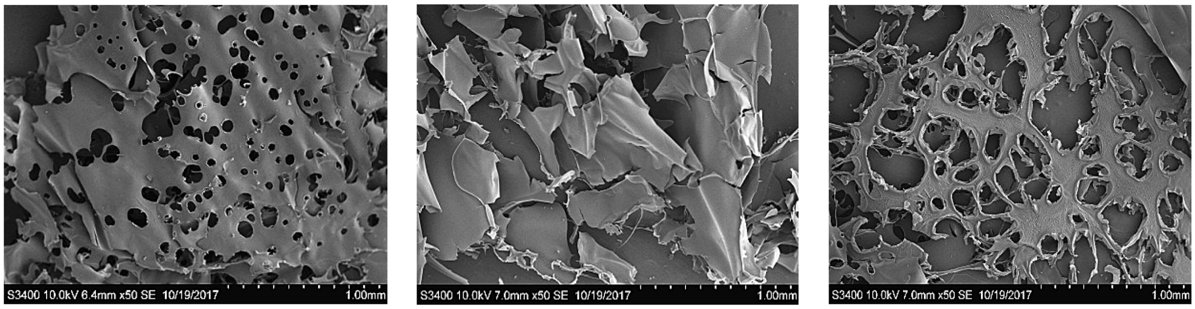
S3400 10.0kV 6.4mm x50 SE 10/19/2017
1.00mm
S3400 10.0kV 7.0mm x50 SE 10/19/2017
1.00mm
S3400 10.0kV 7.0mm x50 SE 10/19/2017
1.00mm

Fig. S4
